# In Vitro Maturation of Bovine Oocytes in the Presence of Resveratrol and Ellagic Acid but Not Chlorogenic Acid Modulates Blastocyst Antioxidant Gene Expression Without Impacting Embryo Development and Oxygen Consumption

**DOI:** 10.3390/antiox14060621

**Published:** 2025-05-23

**Authors:** Katrin Giller, Dominique Schmid, Idil Serbetci, Manuel Meleán, Sarah Greve, Ferdinand von Meyenn, Heinrich Bollwein, Carolina Herrera

**Affiliations:** 1 Department of Molecular Nutritional Science, University of Hohenheim, Garbenstrasse 30, 70599 Stuttgart, Germany; sarah.greve@uni-hohenheim.de; 2 Animal Nutrition, ETH Zurich, Universitaetstrasse 2, 8092 Zurich, Switzerland; 3 AgroVet-Strickhof, University of Zurich, Eschikon 27, 8315 Lindau, Switzerland; idil.serbetci@tarimorman.gov.tr (I.S.); mmelean@vetclinics.uzh.ch (M.M.); heinrich.bollwein@uzh.ch (H.B.); carolina.herrera@uzh.ch (C.H.); 4 Laboratory of Nutrition and Metabolic Epigenetics, ETH Zurich, Schorenstrasse 16, 8603 Schwerzenbach, Switzerland; ferdinand.vonmeyenn@hest.ethz.ch

**Keywords:** polyphenol, antioxidant enzymes, blastocyst rate, hatching rate, developmental competence, dairy cow, embryo metabolism, glutathione peroxidase

## Abstract

In vitro fertilization is used to produce embryos from high-genetic-merit cattle. However, these embryos often exhibit inferior quality than those derived in vivo, possibly due to increased oxidative stress. This study investigates whether adding antioxidant polyphenols (resveratrol (RV), chlorogenic acid (CA), ellagic acid (EA)) to the in vitro maturation (IVM) medium at 0.25, 0.5, and 1 μM could improve embryo development. Oxygen consumption and gene expression were evaluated at the blastocyst stage following treatment with 1 μM of each polyphenol. Embryo development (cleavage, blastocyst, and hatched blastocyst rates) and oxygen consumption were not significantly affected by polyphenols. However, RV significantly upregulated the mRNA expression of the antioxidant enzyme glutathione peroxidase 4 (GPX4), while GPX4 expression was significantly downregulated by EA. Expression of other gene markers related to antioxidant defense, apoptosis, development, and metabolism was not significantly affected. The results indicate that applying RV, CA, and EA during bovine oocyte IVM does not enhance in vitro embryo development at the tested concentrations. Given the opposing effects of RV and EA on the expression of GPX4, the effects of those polyphenols regarding the protection of embryos from oxidative stress and potential long-term effects on the offspring remain to be elucidated.

## 1. Introduction

In recent years, assisted reproductive technologies have advanced significantly and are increasingly being used to produce embryos from high-genetic-merit cows [1]. Among these, in vitro fertilization (IVF) has become a widely utilized approach, allowing for the manipulation and observation of early embryonic development in a controlled laboratory setting. Although in vitro bovine embryo production has improved in recent decades, the quality of embryos produced in vitro still remains inferior to that of embryos derived in vivo [2].

An important step for in vitro embryo production is the in vitro maturation (IVM) of oocytes prior to fertilization. Oocyte maturation involves complex biochemical and molecular events, including cytoplasmic and nuclear changes, which are essential for successful fertilization and subsequent embryo development. The environmental oxygen concentration of in vitro systems is often higher compared to the in vivo situation, likely increasing the concentration of reactive oxygen species (ROS) during the IVM procedure [1,3]. The ROS can accumulate in the cytoplasm of embryos cultured in vitro, thus affecting the outcome of IVF [1,4]. In physiological concentrations, ROS have a regulatory role in reproductive processes such as folliculogenesis, oocyte maturation, and fertilization [5]. In supraphysiological concentrations, ROS can oxidize macromolecules, leading to the inactivation of enzymes, the destruction of cellular and cell organelle (e.g., mitochondria) membranes, and the fragmentation of DNA, ultimately resulting in apoptosis and cell death.

In vivo, embryos are protected from oxidative stress by antioxidants present in reproductive fluids and by the expression of antioxidant enzymes across the female reproductive tract [4,6,7]. In vitro culture (IVC) lacks those enzymatic and non-enzymatic antioxidants, resulting in an imbalance of pro- and antioxidants and, thus, oxidative stress [3]. Therefore, potential ROS-lowering and development-improving effects of supplementing media used in in vitro embryo production with a broad variety of antioxidant compounds are investigated [8].

**Figure 1:**
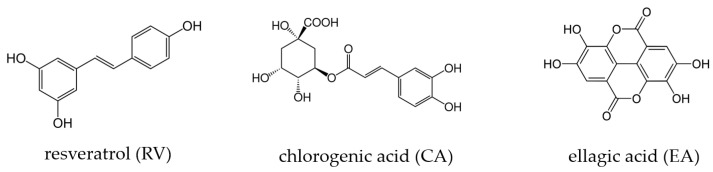
Chemical structures of the polyphenols resveratrol, chlorogenic acid, and ellagic acid used for the pre-incubation of bovine oocytes during in vitro maturation in the present study.

Among natural antioxidant compounds, polyphenols, a group of naturally occurring phytochemicals characterized by several hydroxyl groups (Figure 1), have raised interest for their potential to improve IVM and subsequent embryo development [9].

Previous studies have shown that supplementing IVM media with various polyphenols can benefit oocyte maturation and embryo development, e.g., by increasing intracellular glutathione levels, reducing intracellular ROS, and modulating the expression of genes involved in apoptosis and embryo development [10,11,12,13]. In contrast, polyphenols may also exhibit detrimental effects on embryo development depending on their type and concentration [14,15,16]. The optimal type and concentration of polyphenols for supporting bovine oocyte IVM and embryo development remain unknown. However, adding resveratrol (RV; Figure 1) at 1 and 2 μM to IVM media has been shown to reduce ROS and increase glutathione in matured oocytes, improve blastocyst and hatching rates, and raise total embryonic cell numbers compared to controls [12,17]. To date, the effect of chlorogenic acid (CA; Figure 1) on bovine oocyte IVM has been investigated in only one study [18]. At 5 μM or higher, CA in the IVM medium reduced the maturation and cleavage rate, while no effect was observed at 1.25 μM. Ellagic acid (EA; Figure 1) has not yet been tested in the context of bovine oocyte IVM. However, a recent study on porcine oocytes demonstrated that supplementing IVM media with 10 μM of EA significantly improved embryo developmental competence [19]. The ROS levels were significantly reduced, and glutathione levels increased, while the expression of several antioxidant genes (catalase (CAT), superoxide dismutase 1 (SOD1), heme oxygenase 1 (HO-1), and their upstream transcription factor nuclear factor E2-related factor 2 (NRF2)) was significantly upregulated by EA [19].

This study investigates whether supplementing the IVM medium of bovine oocytes with polyphenols (RV, CA, EA) influences embryo development (cleavage rate, blastocyst rate, and hatched blastocyst rate), metabolic activity (assessed via oxygen consumption rate), and the mRNA expression of key genes associated with oxidative stress and redox regulation (*CAT*, glutathione peroxidase 4 (*GPX4*), thioredoxin (*TXN*)), apoptosis (Bcl-2-associated X (*BAX*)), oocyte development (mitochondrial transcript *FL405*, keratin 8 (*KRT8*)), lipid metabolism (very-long-chain fatty acid elongase 1 (*ELOVL1*)), and cell adhesion (galectin 3 (*LGALS3*)). By elucidating the impact of polyphenols on oocyte maturation and subsequent embryo development, this study aims at contributing to the optimization of IVF protocols, ultimately improving in vitro embryo production for cattle.

## 2. Materials and Methods

### 2.1. In Vitro Production and Culture of Bovine Embryos

Bovine ovaries were retrieved from the slaughterhouse Zurich (SBZ Schlachtbetrieb Zürich AG, Switzerland) and transported in 0.9% NaCl at 38 °C within 2 h to the lab at AgroVet-Strickhof (Lindau, Switzerland). All follicles 2–5 mm were aspirated using a 21 G needle connected to a pump. The follicular fluid containing the cumulus–oocyte complexes (COCs) was collected in a sterile glass bottle containing PBS and maintained at 38 °C. Only COCs with a homogenous cytoplasm and several layers of cumulus cells were selected under a stereomicroscope equipped with a warming plate at 38 °C, separated from other cell types, and placed in BO-Wash (IVF Bioscience, Falmouth, Cornwall, United Kingdom). All selected COCs were placed in groups of 10 in 40 µL microdroplets of bovine in vitro maturation (BO-IVM) medium (IVF Bioscience, Falmouth, Cornwall, United Kingdom) under sterile mineral oil for 22 h in 5% CO_2_, 18.5% O_2_, at 38.5 °C, and saturated humidity. The IVM was performed without (control) or with the addition of polyphenol compounds (RV, CA, EA; all obtained from Sigma-Aldrich Chemie GmbH, Steinheim, Germany) dissolved in dimethylsulfoxide (DMSO; Sigma-Aldrich Chemie GmbH, Steinheim, Germany), resulting in final concentrations of 0.25 μM, 0.5 μM, and 1 μM in the BO-IVM medium. In addition to the control, a DMSO control (Dcon) corresponding to the highest concentration in the polyphenol dilution series was included in each run. Different treatments were performed in separate Petri dishes. Three replicates were performed per polyphenol compound and concentration combination as well as the respective controls.

All IVF runs were performed with frozen semen from the same ejaculate from one bull (Victorinox 193463 D-KBR 002 EWG, 14.08.18, Besamungsverein Neustadt a.d. Aisch e.V., Neustadt/Aisch, Germany). After 22 h of IVM, up to 20 oocytes were placed into 50 μL droplets of IVF medium (BO-IVF, IVF Bioscience, Falmouth, Cornwall, United Kingdom) under sterile mineral oil. Sperm was selected for IVF by means of density gradient centrifugation at 600× *g* for 15 min in BoviPure 80% (Nidacon, Mölndal, Sweden). The pellet was collected and washed once by centrifugation at 600× *g* for 3 min in Hepes-buffered TALP medium. The resulting pellet was placed into a tube with 100 μL of equilibrated IVF medium. Each IVF droplet containing oocytes was inseminated with 9–11 μL of the sperm suspension in order to achieve a final concentration of 2 × 10^6^ sperm/mL (IVF = day 0 (d0)). After incubation for 24 h at 5% CO_2_, 18.5% O_2_, and 38.5 °C, presumptive zygotes were denuded from their cumulus cells using a stripper tip (135 μm, Origio, Ballerup, Denmark). Subsequently, groups of up to 10 presumptive zygotes were placed into droplets of 40 μL of IVC medium (BO-IVC, IVF Bioscience, Falmouth, Cornwall, United Kingdom) under sterile mineral oil and incubated at 5% CO_2_, 6% O_2_, and 38.5 °C. Two days (48 h) after IVF, the cleavage rate was evaluated, the non-cleaved zygotes were removed from the culture drops, and the cleaved embryos were placed back in the incubator with 5% CO_2_, 6% O_2_, and 38.5 °C for further development. Oxygen concentrations of 18.5% during IVM and IVF as well as 6% during IVC were chosen in accordance with the media manufacturer’s recommendations. On d6, d7, and d8 after IVF, blastocyst rate was evaluated. The hatching rate was evaluated on d8 after IVF. All development rates were calculated based on the respective number of oocytes matured. Blastocysts were individually snap-frozen in liquid nitrogen and stored at –80 °C until RNA isolation.

### 2.2. In Vitro Production and Cryopreservation of Embryos for Extracellular Flux Analysis

Three replicates of IVF were performed as described in the previous paragraph. For each replicate, RV, CA, and EA were supplemented at 1 μM during IVM. In addition, a control with the addition of DMSO was included as described above. Cryopreservation was performed on d7, d8, and d9 of IVC using a controlled-rate liquid nitrogen freezer (Freeze Control^®^ CL-5500, CryoLogic Pty. Ltd., Blackburn, Australia). Only expanded (stage code 7), hatching (stage code 8), and hatched (stage code 9) blastocysts of quality codes 1 and 2 were selected according to the International Embryo Transfer Society guidelines [20]. Embryos were stored in liquid nitrogen until thawing for extracellular flux analysis. Selected embryos were washed twice in holding medium (Hepes-buffered TCM-199 with 10% fetal bovine serum) at 25 °C, transferred to cryopreservation medium (ethylene glycol, ABT 360, Pullman, WA, USA), and immediately placed into 0.25 mL straws. After 5 min, the straws were placed into the temperature controller at −7 °C for further 5 min, after which the ice crystal formation was induced by touching the straw with a cotton swab embedded in liquid nitrogen. Embryos were cooled at a rate of 0.6 °C/min. Once the temperature reached −32.2 °C, the straws containing the embryos were immersed in liquid nitrogen and stored until embryo thawing.

### 2.3. Analysis of Blastocyst Real-Time Oxygen Consumption Rate

One day before the measurement of real-time oxygen consumption rate (OCR) by extracellular flux analysis, the straws containing the embryos were thawed for 5 s in the air at room temperature and then immersed in a water bath at 25 °C for 20 s. A dish was prepared containing 500 μL of warming medium (Hepes-buffered TCM-199 with 10% fetal bovine serum and 0.5 M of sucrose) at 38 °C. The contents of the straw were emptied into the dish and further transferred into another well containing 500 μL of the same medium. After 5 min, embryos were transferred to 500 μL of holding medium for another 5 min. Finally, the embryos were placed into 40 μL of pre-equilibrated IVC medium under mineral oil and in vitro cultured in 5% CO_2_ and 6% O_2_ at 38.5 °C until OCR measurement the following day. Survival rates of blastocysts following cryopreservation were 72.1 ± 0.70% for Dcon, 79.0 ± 2.81% for RV, 80.6 ± 0.88% for CA, and 87.1 ± 7.41% for EA. Only re-expanded blastocysts were selected for OCR measurement. Seahorse XFp Extracellular Flux Cartridge utility plates (Aligent Technologies, Waldrom, Germany) were hydrated with sterile water according to the manufacturer’s instructions. The plates as well as the XF calibrant (Agilent Technologies, Waldrom, Germany) were incubated overnight in a CO_2_-free, humidified incubator at 37 °C.

On the day of the measurement, the water was removed from the utility plate, and the pre-warmed XF calibrant was added to the wells and moats as described in the manufacturer’s instructions. The sensor cartridge was lowered onto the utility plate, and the assembled sensor cartridge was incubated for 60 min in the CO_2_-free, humidified incubator at 37 °C before performing calibration in the Seahorse XFp Analyzer (Aligent Technologies, Waldrom, Germany) according to the manufacturer’s instructions.

Groups of five blastocysts per treatment were placed into wells of the Seahorse XFp cell culture miniplates (Agilent Technologies, Waldrom, Germany) containing 180 μL of SOF medium [21], modified without sodium bicarbonate and bovine serum albumin. At least three replicates (one per IVF run) were analyzed for RV, CA, EA, and their corresponding Dcon. To avoid floating of embryos and exclusion from the formed measurement chambers potentially caused by mixing, round grids were cut from 40 μm cell strainers using a 4 mm disposable biopsy punch (KAI Europe GmbH, Solingen, Germany) and were carefully lowered into the wells using sterile tweezers. This ensured the embryos to stay below the grids and thus in the formed measurement chambers when the sensor cartridge was lowered for measurements. Wells containing 180 μL of SOF medium and a grid served as background wells to account for changes in oxygen concentration in the medium independent of the presence of embryos. The pre-warmed plate containing the embryos was loaded into the Seahorse XFp and analyzed via the following protocol: 12 min equilibration followed by six cycles (M1–M6) of 3 min OCR measurement and 3 min re-equilibration via lifting of sensor cartridge and mixing. To calculate the mean OCR, only the most stable measurements (M2–M6) were averaged from the six measurements performed. The output data obtained as OCR in pmol/min/well were normalized to the number of embryos per well.

### 2.4. Isolation of Blastocyst Total RNA and Gene Expression Analysis

Embryos from each of the three 1 μM polyphenol groups and from the Dcon were used for gene expression analysis. The selection of only samples from the 1 μM concentration was made due to limited resources as well as to ensure comparability with the existing literature where the concentration of 1 µM emerges as the most used and consistent across studies. Total RNA was isolated from 6 individual d8 blastocysts per treatment (derived from 3 replicates, n = 2 per replicate) using the RNeasy Micro Kit (Qiagen, Venlo, The Netherlands) according to the manufacturer’s instructions. Concentration of total RNA was determined using a NanoDropTM One/OneC Microvolume UV–Vis spectrophotometer (Thermo Fisher Scientific, Waltham, MA, USA). The RNA integrity was evaluated using the Agilent RNA 6000 Pico Kit (Agilent Technologies, Waldrom, Germany) on a 2100 Bioanalyzer (Agilent, Santa Clara, CA, USA). All samples had an RNA integrity number of at least 5.5. Reverse transcription was performed with 44 ng of total RNA per sample using the GoScript Reverse Transcription System (Promega AG, Dübendorf, Switzerland) according to the manufacturer’s instructions (25 °C for 5 min, 42 °C for 60 min, 70 °C for 15 min).

The KAPA Sybr Fast Mix (Kapa Biosystems, Wilmington, NC, USA) was used according to the manufacturer’s instructions to perform quantitative real-time polymerase chain reaction on a LightCycler instrument (System LightCycler 96, Roche Diagnostics GmbH, Basel, Switzerland) with 40 cycles of amplification (95 °C for 10 s, 60 °C for 20 s) and the following melting conditions: 95 °C for 5 s, 65 °C for 60 s, 97 °C for 1 s. A single threshold was applied to obtain the cycle of quantification (Cq) values. Primers (Table 1) were ordered from Microsynth (Balgach, Switzerland). According to the geNorm output for the three reference genes tested (actin beta (*ACTB*), H3.3 histone A (*H3-3A*), and tyrosine 3-monooxygease/tryptophan 5-monooxygenase activation protein zeta (*YWHAZ*)), the geometric mean of the Cqs of ACTB and H3-3A was used to calculate relative expression levels for each target gene. The relative expressions of the eight target genes (*BAX, CAT, ELOVL1, FL405, GPX4, KRT8, LGALS3*, and *TXN*) in the polyphenol groups were compared to those of the Dcon group according to Livak and Schmittgen [22].

### 2.5. Statistical Analysis

Data were analyzed using R Version 4.3.2 (R core Team, 2023). Normal distribution of the residuals was determined by the Shapiro–Wilk normality test. In the RV group, cleavage rate data were not normally distributed and thus analyzed using the Kruskal–Wallis test. All other embryo development rates and OCR data followed a normal distribution. These data were analyzed for differences by one-way analysis of variance (ANOVA). Effects of the polyphenols on gene expression were determined for normally distributed data by two-sample *t*-test (all except: RV: KRT8 and LGALS3; EA and CA: TXN) or by Mann–Whitney U test for non-normally distributed data. For all statistical analyses, the effect of the IVF batch was excluded by computing Likelihood Ratio Tests. No significant differences in embryo development rates were observed between control and Dcon (Table A1), but, to still account for a potential small influence of DMSO on the parameters, OCR measurements and qPCR were performed only with Dcon as control group. In the following, Dcon will thus be referred to as “control”. A *p* ≤ 0.05 was considered significant, while 0.05 < *p* < 0.1 was considered a trend.

## 3. Results

### 3.1. In Vitro Maturation in the Presence of Polyphenols Did Not Affect Embryo Development

**Figure 2:**
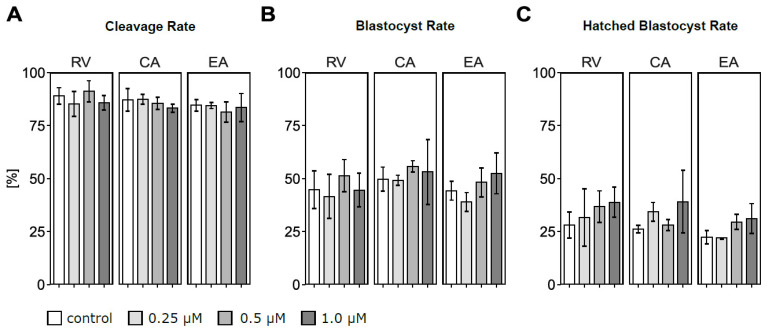
Effects of adding polyphenols (resveratrol (RV), chlorogenic acid (CA), ellagic acid (EA)) in concentrations of 0.25, 0.5, and 1.0 μM to the in vitro maturation medium of bovine oocytes on cleavage, blastocyst, and hatched blastocyst rates determined on d2, d6–8, and d8 after in vitro fertilization, respectively: (**A**) Effects of polyphenols on the cleavage rate. (**B**) Effects of polyphenols on the blastocyst rate. (**C**) Effects of polyphenols on the hatched blastocyst rate. Each data point refers to an initial number of 16–33 oocytes. Exact numbers of oocytes matured per treatment group as well as the total numbers of cleaved embryos, blastocysts, and hatched blastocysts obtained across all replicates are presented in Table A2.

Total oocyte numbers used for maturation across three biological replicates were 82–84 in RV, 69–70 in CA, and 82–84 in EA batches. Cleavage, blastocyst, and hatched blastocyst rates did not significantly differ according to type and concentration of polyphenol treatment compared to their respective control group (Figure 2A–C). Nevertheless, a high variability in hatched blastocyst rates was observed, with hatched blastocyst rates always numerically higher in the 1 μM of polyphenol compared to the control group. With 1 μM of RV supplementation, the average hatching rate was 38.8% while it was 28.1% in the control group (Figure 2C). Similarly, incubation with 1 μM of CA numerically increased (39.1%) the hatching rate compared to controls (26.2%), and 1 μM of EA resulted in a 31.2% hatching blastocyst rate compared to 22.3% in the respective control group.

### 3.2. Polyphenol Supplementation During In Vitro Maturation Did Not Affect Blastocyst Oxygen Consumption Rate

**Figure 3:**
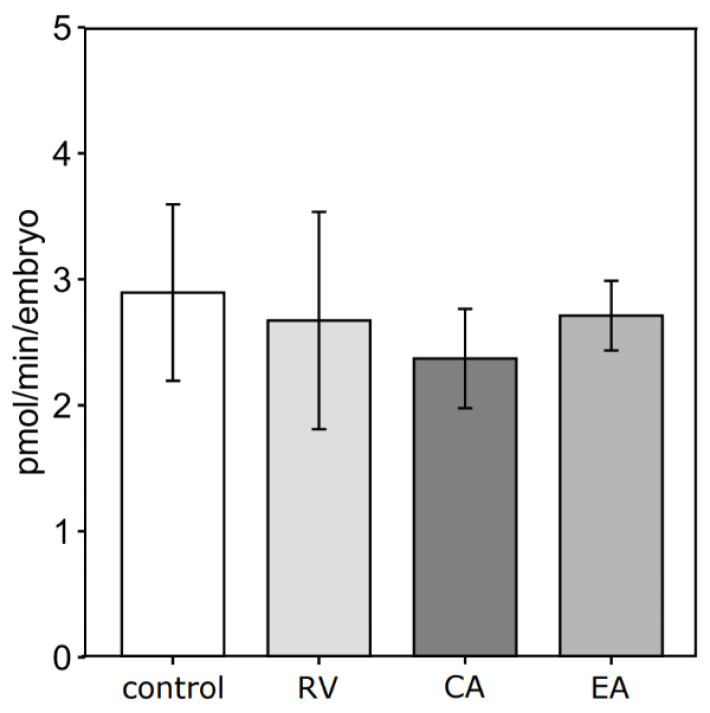
Effects of adding 1.0 μM of resveratrol (RV, n = 3), chlorogenic acid (CA, n = 4), or ellagic acid (EA, n = 3) to the in vitro maturation medium of bovine oocytes on real-time oxygen consumption rate of d8 blastocysts compared to their unsupplemented control (n = 3). Each well contains groups of 5 blastocysts, and 5 consecutive measurements at intervals of 6 min each are performed per well.

For OCR measurements, only blastocysts from the 1 μM polyphenol groups and the respective control group were used. No significant differences in d8 blastocyst OCRs were observed among the experimental groups (Figure 3). The numerically lowest mean OCR of 2.37 pmol/min per blastocyst was detected for CA, while control blastocysts showed the numerically highest mean OCR of 2.89 pmol/min. Incubation with RV and EA at 1 μM resulted in intermediate mean blastocyst OCR values of 2.67 and 2.71 pmol/min, respectively.

### 3.3. Blastocyst mRNA Expression of Genes Involved in Antioxidant Defense Was Affected by In Vitro Maturation in the Presence of Ellagic Acid and Resveratrol

**Figure 4:**
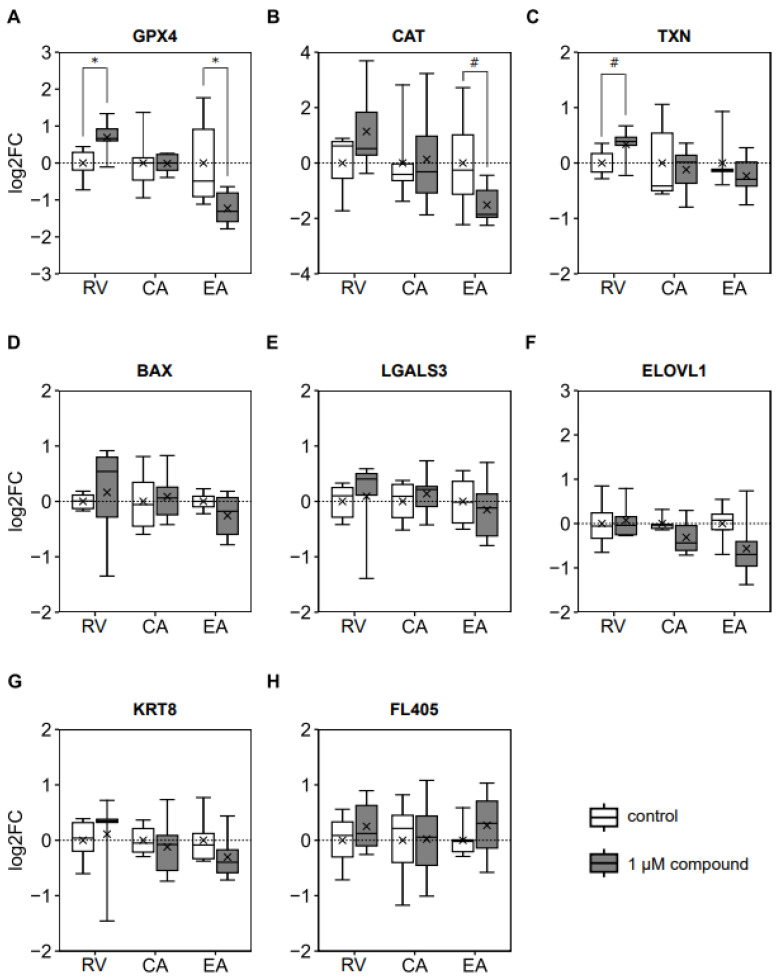
Effects of adding 1.0 μM of resveratrol (RV), chlorogenic acid (CA), or ellagic acid (EA) to the in vitro maturation medium of bovine oocytes on the relative mRNA expression of d8 blastocysts (n = 6 per group): (**A**) Catalase (CAT). (**B**) Glutathione peroxidase 4 (GPX4). (**C**) Thioredoxin (TXN). (**D**) Bcl-2-associated X (BAX). (**E**) Galectin 3 (LGALS3). (**F**) Very-long-chain fatty acid elongase 1 (ELOVL1). (**G**) Keratin 8 (KRT8). (**H**) Mitochondrial transcript FL405. * *p* < 0.05; # 0.05 < *p* < 0.1.

Oocyte maturation in the presence of RV increased (*p* = 0.027), while EA reduced (*p* = 0.046) blastocyst gene expression of GPX4 (Figure 4A). Similarly, the addition of EA to the IVM medium tended to decrease (*p* = 0.087) blastocyst CAT expression (Figure 4B). Adding RV to the IVM medium tended to increase (*p* = 0.062) blastocyst TXN gene expression (Figure 4C). The expression of all other analyzed genes (BAX, LGALS3, ELOVL, KRT8, and FL405) was not affected by incubation with polyphenols at concentrations of 1 μM during IVM (Figure 4D–H).

## 4. Discussion

This study is the first to investigate the effects of supplementing bovine oocytes with EA during IVM on subsequent embryo development. The impact of CA on bovine embryo development has been reported only once and solely in the context of supplementation during both IVM and IVC phases [18]. In contrast, RV has been more extensively studied, with several reports investigating its addition to the IVM medium of bovine oocytes [12,17,23]. Therefore, the RV was included in the present study as a reference compound to benchmark the effects of EA and CA, for which no previous data exist regarding supplementation exclusively during IVM of bovine oocytes.

The oral bioavailability of polyphenols is very low in ruminants due to the microbial metabolism of dietary polyphenols in the rumen in addition to their rapid metabolism by phase I and II enzymes and elimination via urine in vivo [24]. Even after intraduodenal application, polyphenol plasma levels are in the nanomolar range in ruminants [24]. Therefore, it is likely that in vivo follicular fluid levels of polyphenols are much lower than the micromolar concentrations used in the present study. Still, the data presented herein are of relevance for the process of in vitro embryo production when media is supplemented with antioxidants to ameliorate the increased oxidative stress compared to the in vivo situation [1,3,8].

### 4.1. Embryo Development

Embryo development, as measured by cleavage, blastocyst, and hatched blastocyst rates, was unaffected by 0.25, 0.5, or 1 μM of RV in this study, contrasting a previous report showing improved blastocyst (30% vs. 22%) and hatched blastocyst (31% vs. 15%) rates with 1 μM of RV during bovine oocyte IVM [17]. Although not statistically significant, the numerical difference in hatched blastocyst rate in the present study (39% vs. 28%; +11%) was similar to the +16% improvement reported by Wang et al. [17]. Both studies found no effect of 1 μM of RV on cleavage rates, and neither low (0.1 μM) nor high (10 μM) RV concentrations significantly affected embryo development [17]. The present results are in line with Torres et al. [25], who did not report differences in cleavage or blastocyst rates using 1 or 10 μM of RV (as a cyclodextrin complex) during bovine oocyte IVM. Sovernigo et al. [12] found a modest increase in blastocyst rate with 2 μM of RV (54% vs. 47%) in bovine oocyte IVM medium, with no effect on hatching. Other concentrations were not tested. In aged bovine oocytes, 2 μM of RV in IVM medium did not improve blastocyst rates, suggesting that RV could not reverse age-related declines in oocyte competence [26].

Supplementing IVM media with RV at 0.25–5 μM has been reported to improve oocyte quality and embryo development across multiple species, including mouse, cat, pig, goat, sheep, buffalo, and human [11,27,28,29,30,31,32,33]. In ruminants other than cattle (goat, sheep, buffalo), embryo development improved with 0.25–1 μM of RV, while higher concentrations (≥2 μM) had no effect or were detrimental; for example, 5 μM of RV impaired development in sheep [30,32,33]. These results thus contrast with the present findings of unaffected bovine embryo development following IVM with 0.25–1 μM of RV. Contrary to ruminants, RV concentrations of 2–5 μM in IVM media were required to enhance embryo development in the non-ruminant species pig and cat, with lower concentrations of 0.1–1 μM showing no effect [11,27,29,31]. Still, in mice and humans, 1 μM of RV during IVM did enhance embryo development [28].

Supplementing RV beyond a certain, potentially species-specific, threshold (e.g., 5 μM of RV in sheep as mentioned above) seems to negatively affect embryo development. In cattle, this threshold appears to be higher since 10 μM of RV during bovine oocyte IVM did not impair the blastocyst rate [17]. With 20 μM of RV during bovine oocyte IVM, Takeo et al. [34] observed an increased blastocyst total cell number but did not report blastocyst rates. However, RV concentrations of 20 and 40 μM strongly reduced bovine oocyte maturation rates compared to controls (57 and 35 vs. 84%) [14], suggesting that high concentrations may impair oocyte competence.

A key aspect to consider with regard to the present study is the use of commercial media for all stages of in vitro embryo production, meaning that the exact composition is unknown. As commercial media often contain antioxidants, it cannot be ruled out that the IVM medium contained RV, which is the most studied polyphenol among those used in the present study. In that case, the actual RV concentrations may have exceeded the intended levels.

To date, only one study has examined CA supplementation in bovine in vitro embryo production, but only in both IVM and IVC media [18]. While 10 and 20 μM of CA reduced oocyte maturation and embryo development, lower concentrations (1.25–5 μM) showed no significant effects. In the present study, 1 μM of CA did not significantly affect the hatched blastocyst rates compared to the control (39% vs. 26%), suggesting that CA concentrations in IVM medium below 1.25 μM are safe, whereas higher levels may impair developmental competence. In contrast, porcine embryos tolerated much higher CA levels. Supplementation with 50 μM of CA during IVM either improved or did not affect maturation, fertilization, and blastocyst rates [35,36,37,38], and even concentrations of 100–200 μM were not detrimental [35,36]. Interestingly, 50 μM of CA in porcine oocyte IVM medium enhanced blastocyst formation in parthenogenetic embryos but not somatic cell nuclear transfer embryos [39]. These findings highlight potential species-specific responses to the type and concentration of polyphenols during IVM, emphasizing the need for cross-species studies under standardized conditions.

As no prior studies have examined EA supplementation during bovine oocyte IVM, the present data provide a foundation for future research. In a recent study, 10 μM of EA in porcine oocyte IVM medium improved cleavage and blastocyst rates after parthenogenetic activation [19], and 20 μM of EA in IVF medium increased porcine blastocyst yield [40]. The considerably lower concentrations of EA used in the present study (≤1 μM) may explain the lack of observed effects. However, based on bovine and porcine data for RV and CA, higher EA concentrations could potentially be detrimental, warranting further investigation.

Laboratory-specific efficiency in in vitro embryo production may also influence outcomes. Labs with lower baseline development rates might benefit more from polyphenol supplementation. However, control cleavage (85–89% vs. 70–86%), blastocyst (44–50% vs. 20–51%), and hatched blastocyst rates (22–28% vs. 15–63%) in the present study (Table A1) were comparable to those reported in previous RV and CA studies [12,17,18,23].

The solvent used for polyphenol stock solutions may also play a role. While Wang et al. [17] did not specify a solvent, others used methyl β-cyclodextrin in acetone/water [23] or DMSO [12,18]. Even though Pérez-Pastén et al. [41] reported an embryo-protective, free radical scavenging effect of DMSO in the IVC of rat embryos against hydroxyurea-induced damage, DMSO alone showed no significant effect on the blastocyst formation rate in mice when applied during IVC [42] as well as on embryo development in the present study (Table A1).

### 4.2. Blastocyst Oxygen Consumption

The present study is the first to assess the effects of polyphenol supplementation during oocyte IVM in general, and bovine oocytes in particular, on the OCR of in vitro produced embryos. Mitochondria in oocytes and embryos consume oxygen to generate ATP, which supports maturation and development [29]. As bovine embryos progress from the 2–4 cell stage to the blastocyst stage, mitochondrial activity and thus OCR increase significantly [43,44].

In the present study, mean OCRs of blastocysts (measured in groups of five) ranged from 2.4 to 2.9 pmol/min per blastocyst. These values exceed the average OCR reported by Overstrom et al. [45] for individually measured in vivo-derived bovine blastocysts (~1.1 pmol/min, range: 0–7.3 pmol/min), and also those by Muller et al. [44] (~0.85 pmol/min), who used the same Seahorse XFp Analyzer as the present study and analyzed six blastocysts per well.

The variability in OCR might be due to the age of the blastocysts that were measured on d6–8 by Muller et al. [44], on d7 by Overstrom et al. [45], and on d8 in the present study, thus further demonstrating an increasing OCR with increasing embryo age even across studies. Furthermore, Overstrom et al. [45] used in vivo-derived embryos, while the embryos analyzed by Muller et al. [44] and in the present study were produced in vitro. The increased exposure to atmospheric O_2_ in vitro compared to in vivo increases embryo glycolytic activity and pyruvate utilization [1], and this differently adjusted metabolism likely affects the blastocyst OCR.

Analyzing blastocysts can be additionally challenging due to their varying stages, such as early, expanded, hatching, or hatched, despite being classified as blastocysts. In line with the increasing mitochondrial activity during embryonic development, Muller et al. [44] noted a trend of higher OCRs in expanded versus early blastocysts. In the present study, it was not possible to use five blastocysts at the same developmental stage per well due to a lack of identical-stage blastocysts per treatment. Therefore, each well contained three hatched and two hatching blastocysts, with this distribution monitored after measurements to ensure that hatching was not completed during the analysis. A human study showed that metabolic activity differs between in vitro produced blastocyst portions inside and outside the zona pellucida [46], potentially introducing bias in this measurement. Ideally, individual embryos would be analyzed to avoid this bias, but the Seahorse XFp Analyzer used in this study allowed the reliable measurement of a minimum group of five blastocysts, which is already slightly fewer than the six per well analyzed by Muller et al. [44].

In addition to ATP production, mitochondrial respiration generates ROS [47]. The higher metabolic activity of embryos thus results in more ROS, potentially causing oxidative damage and reducing embryo viability compared to “quiet” embryos with lower energy demands [1]. Therefore, antioxidant polyphenols in in vitro embryo production media may permit enhanced mitochondrial function without increasing oxidative stress [28]. This would reduce the adverse effects of a high metabolic rate in embryos and thus improve their ability for successful implantation and development into a live offspring. To achieve this, a promotion of embryo metabolic activity by the polyphenols must be avoided, and, indeed, 1 μM of RV, CA, or EA did not affect the OCR of d8 blastocysts. It must be considered though, that the blastocysts had undergone cryopreservation and thawing before OCR measurements. A diminishing effect of these processes on potential polyphenol-induced differences in blastocyst OCR cannot be excluded with certainty.

### 4.3. Blastocyst Gene Expression

From the unchanged OCR and embryo development rates, one would expect a similar ROS production and thus a similar activation of antioxidant defense mechanisms, including the expression of antioxidant enzymes. This was not the case since the expression of the antioxidant gene GPX4 was significantly modulated by RV and EA, and this occurred in opposite directions. The first line of defense against ROS is SOD, which reduces O_2_^•−^ to O_2_ and H_2_O_2_. The GPX and CAT then individually reduce H_2_O_2_ to O_2_ and H_2_O [48,49]. The GPX has been identified as crucial for oocyte and early embryo development [1]. This enzyme relies on glutathione, an endogenous non-enzymatic antioxidant, to mitigate oxidative damage and protect against ROS-induced stress [50]. The upregulation of GPX4 expression following RV treatment in the present study suggests the enhanced protection of blastocysts against ROS-induced damage [48]. Previous studies have shown similar effects, with 1 μM of RV upregulating GPX4 expression in bovine cumulus cells [17] and MII-stage murine oocytes [28]. In porcine oocytes treated with 5 μM of RV, CAT expression was significantly upregulated [29], indicating that a higher RV concentration might have been required to enhance CAT expression in the present study. If determined, these changes were accompanied by reduced ROS and increased glutathione levels and, as described above, improved embryo development rates [17,29].

Other polyphenols, like epigallocatechin-3-gallate (50 μM in bovine oocytes; GPX4, CAT, and SOD1) and quercetin (10 μM in mouse oocytes; GPX4), also upregulated the expression of antioxidant enzymes and improved embryo development when applied in IVM media [48,50]. While no significant differences in embryo development were observed in the present study, oxidative damage and ROS formation were not assessed, and these factors may still have varied between treatments and the control group.

The expression of GPX4 is induced upon nuclear translocation of the redox-sensitive transcription factor NRF2 that also induces the expression of further antioxidant enzymes such as NADPH-quinone oxidoreductase, glutathione-S-transferase, glutathione reductase, and SOD [51]. The NRF2 has been shown to be activated by a wide range of polyphenols [52]. Therefore, it is plausible that RV might have triggered NRF2 activation, promoting the expression of further antioxidant enzymes that have not been examined in the present study.

Unlike RV, incubation with EA significantly reduced the expression of GPX4, a finding that was unexpected given that EA, like RV, functions as both a radical scavenger and an NRF2 activator [53]. Based on these shared properties, a similar effect would have been anticipated. One possible explanation is that EA’s higher number of hydroxyl groups might confer a greater intrinsic radical scavenging capacity than RV, potentially reducing the embryo’s need to upregulate NRF2 and its downstream antioxidant enzymes. However, when comparing RV and EA using a chemical assay (Trolox-equivalent antioxidant capacity), both showed similar antioxidant capacities (10.5 and 9.4 µmol of Trolox equivalents/mg, respectively) [54]. Interestingly, in an ex vivo biological assay, RV appeared more effective than EA, requiring a much lower concentration (12 vs. 45 µg/mL) to achieve 50% inhibition of oxidative damage to red blood cell membranes caused by UV exposure [54]. This suggests that RV may exert a stronger antioxidant effect in biological systems, aligning with the current study’s findings. It is also important to note that EA’s antioxidant activity is influenced by environmental factors that affect its structure such as pH and solvent polarity. In solvents capable of forming hydrogen bonds with EA, like DMSO used in the present study, the EA’s antioxidant capacity can be diminished due to interference with its hydrogen atom transfer mechanism [53]. While this same mechanism also applies to RV [55], EA may be more susceptible to such interference. Additionally, polyphenols can exhibit pro-oxidant properties under certain conditions [56], which might have been the case for EA in the present study, though without impairing embryo development.

The CA did not affect antioxidant gene expression in the present study. This is notable given that the previously mentioned studies supplementing CA to IVM and IVC media did not assess gene expression [18,35,36,37,38]. In principle and just like other polyphenols, CA is known to be capable of inducing *NRF2* translocation and activating the downstream expression of antioxidant enzymes [57].

In contrast to the effects of RV and EA on GPX4 expression, the expression of TXN and CAT was not significantly affected. This differential response among antioxidant defense genes suggests gene-specific regulation and sensitivity to polyphenol-induced modulation during IVM. Similarly, a previous study in maturing rat oligodendrocytes reported two- to threefold increased GPX expression at constant CAT expression [58]. The threshold for modulating CAT expression thus appears to be higher than that for modulating GPX4 expression [58]. This may explain why RV and EA significantly affected GPX4 but not CAT expression. A homozygous knockout of GPX4 in mice resulted in the intrauterine death of offspring between days 7.5 and 8.5 [59,60]. In contrast, the homozygous knockout of CAT was not detrimental to intrauterine embryo survival in mice [61]. Therefore, it can be concluded that GPX4 is essential for early embryo development while CAT is not, which may be reflected in their responsiveness to environmental stimuli.

As for GPX4, the transcription of CAT and TXN is also regulated by NRF2 [62,63]. However, while GPX4 and CAT expressions are additionally induced by FOXO, the latter has not been described as a transcription factor for TXN [51,62]. The unaffected TXN expression may thus indicate that TXN requires a different additional type of redox stimulus to be transcriptionally activated. The present findings therefore indicate a selective sensitivity of the embryonic antioxidant system to dietary polyphenols.

In line with the similar OCR across treatment groups, indicating similar mitochondrial metabolism, the mitochondrial transcript FL405 and the pro-apoptotic BAX were not significantly regulated by the polyphenol treatments. Since BAX is involved in mitochondria-mediated apoptosis and has been associated with reduced oocyte quality and survival [63,64], its downregulation is generally considered indicative of improved embryo quality [65]. However, in this study, no significant changes in BAX expression were observed. This outcome contrasts with findings from other studies where polyphenols such as RV (2 μM), epigallocatechin-3-gallate (10 μM), and trans-ε-viniferin (0.5 μM) in porcine IVM media, as well as 3,4-dihydroxyflavone (10 μM) in bovine IVC medium reduced BAX expression in cumulus cells, matured oocytes, and blastocysts [11,64,66,67]. The lack of effect in the current study may be attributed to the 1 μM concentration used. Supporting this, Lee et al. [16] also reported no change in BAX expression in porcine blastocysts produced and cultured with 0.5 μM of RV. While other apoptosis-related genes like BCL2 and caspase-3 have shown decreased expression in response to RV (0.5–2 μM) in porcine oocytes and blastocysts [11,16], these markers were not included in the current gene panel and therefore remain unassessed.

In addition to antioxidant and apoptotic markers, the present study investigated the expression of genes that have previously been identified as potential embryonic indicators for successful pregnancy. Specifically, a higher expression of FL405 and LGALS3 has been linked to failed pregnancies or pregnancy loss, while a higher expression of ELOVL1, KRT8, and TXN has been associated with successful calf delivery [68,69]. The absence of significant changes in the expression of these genes in bovine blastocysts resulting from oocytes incubated with polyphenols during IVM aligns with this study’s findings of unchanged OCR and embryo development rates.

A noteworthy consideration is the potential influence of embryo sex on gene expression. For instance, KRT8 has been shown to be expressed at higher levels in female compared to male bovine embryos [68]. Since the sex of the embryos was not determined in the present study, variability in the male-to-female ratio could have introduced bias into the gene expression results. This highlights the importance of considering sex as a variable in transcriptomic analyses, particularly when evaluating genes with known sex-dependent expression patterns.

Finally, it must be acknowledged that the present study randomly used hatched and non-hatched embryos for gene expression analysis. This could have additionally affected the gene expression results, as previous work by Chen et al. identified 85 genes that were differently expressed between non-hatched and hatched mouse blastocysts [70]. Such stage-dependent transcriptional differences suggest that uniform embryo selection, based on developmental stage, may be critical for accurately interpreting gene expression outcomes.

## 5. Conclusions

The absence of significant differences in embryo development rates and the OCR of blastocysts following IVM with RV, CA, and EA at 0.25, 0.5, and 1 μM indicates that these polyphenols and concentrations are safe to be applied in bovine in vitro embryo production, but without clear benefits. The reduced expression of antioxidant GPX4 by EA requires further attention though. Whether polyphenols impact embryo development when applied not only during IVM but also during IVF and/or IVC remains unknown. With thousands of polyphenol structures largely unexplored in this context, their full potential for in vitro embryo production is far from realized. Importantly, studies in different species show species-specific sensitivity of oocytes and embryos towards different polyphenol concentrations. Species-specific responses to polyphenols therefore need clarification through comparative studies using the same treatment across different species. Future research should use well-defined media of known composition and consider embryo sex and developmental stage to enhance data reliability and comparability.

## Figures and Tables

**Table 1:** Sequences of the primers used for qPCR.

Gene	Accession Number	Forward Primer (5′-3′)	Reverse Primer (5′-3′)	Amplicon Length (bp)
*ACTB*	NM_173979.3	GATCTGGCACCACACCTTCT	AGAGACAGCACAGCCTGGAT	174
*BAX*	NM_173894.1	GCCCTTTTGCTTCAGGGTTT	ACAGCTGCGATCATCCTCTG	179
*CAT*	NM_001035386.2	CTGGGACCCAACTATCTCCA	AAGTGGGTCCTGTGTTCCAG	179
*ELOVL1*	NM_001034703	GTACTTCGTCCTCTCACTGG	GCCAGGTGTAGGAACTTAGCC	158
*FL405*	AY308069.1	ACCAGCTCAATCTGCCTCCG	CCGTGGGCAATCATAAGGGC	146
*GPX4*	NM_174770.4	ACCCTCTGTGGAAATGGATG	GAAGGCTTCTCGGAACACAG	228
*H3-3A*	NM_001014389.2	GTACTGTGGCACTCCGTGAA	GATAGGCCTCACTTGCCTCC	168
*KRT8*	NM_001033610.1	GAATGTGCCTTATGACCTGCC	GGCTGTAGTTGAAGCCAGGG	179
*LGALS3*	NM_001102341.2	GAATGTGCCTTATGACCTGCC	TGGAAGGCGACATCATTCCC	132
*TXN*	NM_173968.3	ATCGATTGCACTGTCAGGTCGC	TCTCTCCTGCACTGTTCAAGGC	106

## Data Availability

The raw data supporting the conclusions of this article will be made available by the authors upon request.

## Appendix A

**Table A1:** Embryo development rates in the control groups without and with DMSO.

	Cleavage Rate (%)	Blastocyst Rate (%)	Hatched Blastocyst Rate (%)
control	88.9 ± 3.81	48.3 ± 3.26	33.6 ± 3.97
DMSO control	86.9 ± 1.29	46.2 ± 1.74	25.5 ± 1.72
*p*-value	0.647	0.603	0.135

**Table A2:** Total numbers of oocytes matured per treatment group and total numbers of resulting cleaved embryos, blastocysts, and hatched blastocysts.

	Treatment	Oocytes	Cleaved Embryos	Blastocysts	Hatched Blastocysts
RV	control	84	75	38	24
	0.25 µM	83	71	35	27
	0.5 µM	82	75	42	30
	1 µM	83	71	37	32
CA	control	70	61	34	18
	0.25 µM	70	61	35	25
	0.5 µM	70	60	39	20
	1 µM	69	57	34	24
EA	control	84	71	37	19
	0.25 µM	82	69	32	18
	0.5 µM	83	67	39	24
	1 µM	82	68	42	25
